# O2.3. AUTOMATED ANALYSIS OF RECENT-ONSET AND PRODROMAL SCHIZOPHRENIA

**DOI:** 10.1093/schbul/sby015.193

**Published:** 2018-04-01

**Authors:** Guillermo Cecchi, Cheryl Corcoran

**Affiliations:** 1 IBM Research; 2 Icahn School of Medicine at Mount Sinai

## Abstract

**Background:**

Psychosis has significant effects on language, to the extent that its disturbance is one of the principal components of diagnosis and prognosis. In particular, two features of language seem to be prominently affected: discourse coherence, observed in patients as derailment (or tangentiality), and discourse richness, observed as poverty of speech. Using automated linguistic analysis on baseline interviews, we have shown in a previous study that it is possible to predict with high accuracy of conversion to psychosis (100%) among a cohort of clinical high-risk youth, by quantifying the subjects’ semantic coherence and syntactic complexity as proxies for derailment and poverty of speech, respectively.1 In the present study, we seek to explore to what extent the prodromal prediction model can discriminate recent-onset schizophrenia patients from matching controls, with the intent of understanding how the prodromal-onset transition is reflected in language.

**Methods:**

Eighteen recent-onset schizophrenic patients and twelve matching controls had baseline interviews, using an open-ended protocol previously introduced.2 Using automated analysis, transcripts of interviews were evaluated for semantic and syntactic features predicting psychosis onset in an independent cohort. These features were then used to discriminate between patients and controls, applying the same classifier that predicted conversion to psychosis, namely converts laying outside the convex hull of non-converts. Additionally, we compared the discrimination power of this approach against alternative models including alternative linguistic features (e.g. metaphoricity3) and protocols (e.g. short prompts4).

**Results:**

The convex hull of the controls subjects misclassifies only one of the patient samples, a result that amounts to 95% true positive rate. Surrogating by label randomization and accounting for false and positive negative rates results in a balanced accuracy of 80%, which is comparable to those obtained with alternative automated models, which range from 70% to 85%. Moreover, the present cohort is clearly separable from both converts and non-converts in the CHR cohort when projected in the feature space.

**Discussion:**

The automated features optimized for prediction of psychotic onset convey highly significant information regarding the discrimination between recent-episode patients and controls. The directionality of effects, however, is not obviously derived from that observed in the prodromal-onset transition. This preliminary study provides the basis for a larger study to better understand language disturbances across these cohorts.

**References:**

1.Bedi, G., et al. npj Schizophrenia 1 (2015): 15030.

2. Ben-David, S., et al. Psychiatric Services 65.12 (2014): 1499–1501.

3. Gutiérrez, E.D., et al. Proceedings of the 2017 Conference on Empirical Methods in Natural Language Processing. 2017.

4. Mota, N.B., et al. PloS one 7.4 (2012): e34928.

